# Pulmonary involvement in newly diagnosed and untreated rheumatoid arthritis and psoriatic arthritis: a prospective longitudinal study

**DOI:** 10.1007/s00296-024-05751-w

**Published:** 2024-12-18

**Authors:** Lone Winter, Simon M. Petzinna, Dirk Skowasch, Carmen Pizarro, Marcel Weber, Daniel Kütting, Charlotte Behning, Claus-Jürgen Bauer, Valentin S. Schäfer

**Affiliations:** 1 Department of Rheumatology and Clinical Immunology, Clinic of Internal Medicine III, University Hospital Bonn, Bonn, Germany; 2 Department of Cardiology, Angiology, and Pulmonology Clinic of Internal Medicine II, University Hospital of Bonn, Bonn, Germany; 3 Department of Radiology, University Hospital of Bonn, Bonn, Germany; 4 Institute of Medical Biometry, Informatics and Epidemiology, University Hospital of Bonn, Bonn, Germany; 5 Department of Rheumatology and Clinical Immunology, Clinic of Internal Medicine III, University Hospital of Bonn, Venusberg-Campus 1, 53127 Bonn, Germany

**Keywords:** Autoimmune Diseases, Rheumatoid Arthritis, Psoriatic Arthritis, Lung Diseases, Thoracic Radiography, Follow-up Studies

## Abstract

**Objectives:**

To longitudinally assesses pulmonary involvement in newly diagnosed rheumatoid arthritis (RA) and psoriatic arthritis (PsA) patients over a 12-months follow-up. To identify biomarkers and establish a diagnostic algorithm for monitoring pulmonary changes.

**Methods:**

Newly diagnosed RA and PsA patients were examined with clinical and laboratory assessments, pulmonary function tests (PFT), and chest radiography (CXR) at three-months intervals for one year.

**Results:**

The study enrolled 50 patients (26 RA, 24 PsA) and 26 controls. At baseline, 37.0% of arthritis patients (50.0% RA, 22.7% PsA) exhibited radiographic pulmonary involvement, with 64.7% being asymptomatic. No association was observed between CXR and PFTs. Reduced pathological breathing width was noted in 64.0% of patients (RA 69.2%, PsA 58.3%) and 23.1% of controls (*p* < .001). Thoracic excursion and lung auscultation showed no differences. During follow-up, PFT and physical examination findings remained stable. Mean CRP levels significantly decreased in RA patients from 23.5 mg/l (± 33.6; 95% CI: 9.9–37.1) to 2.7 mg/L (± 3.4; 95% CI: 1.0-4.3), and in PsA patients from 13.3 mg/L (± 18.0; 95% CI: 5.7–20.9) to 8.1 mg/L (± 16.2; 95% CI: 0.1–16.2) (*p* < .001). Additionally, significant reductions in disease activity scores and improvements in six-minute walking distance were observed (*p* < .001). No associations were identified between PFT outcomes, disease activity, or rheumatological medications throughout the disease course.

**Conclusion:**

Our study underscores the prevalence of significant, predominantly asymptomatic pulmonary involvement in newly diagnosed RA and PsA patients. The lack of correlation between pulmonary function, disease activity, and medication during disease progression suggests that reducing arthritic disease activity does not necessarily mitigate the risk or severity of pulmonary involvement. Finally, our finding underscore the need for more sensitive biomarkers and optimized monitoring strategies.

**Supplementary Information:**

The online version contains supplementary material available at 10.1007/s00296-024-05751-w.

## Introduction

Rheumatoid arthritis (RA) and psoriatic arthritis (PsA) are primarily characterized by joint inflammation but frequently involve extra-articular manifestations that can significantly affect patients’ overall health and quality of life [1–4]. Among these, pulmonary involvement stands out as a major contributor to morbidity and mortality [5–7]. Particulary in RA, pulmonary involvement ranks as one of the leading causes of death [5, 8] and is associated with a threefold increase in mortality rates [9–13]. Pulmonary manifestations in RA encompass a wide spectrum of conditions, commonly classified into interstitial lung disease associated with RA (RA-ILD) and non-RA-ILD manifestations [14, 15].

RA-ILD is a chronic and often progressive pulmonary complication frequently encountered in patients with RA [3, 5, 9], marked by hetereogenous structural and functional alterations in the lungs, including pulmonary fibrosis and restrictive lung disease. It results from widespread damage to the lung interstitium, affecting the alveoli, blood vessels, and lymphatic system [14, 16, 17]. Lifetime risks of RA-ILD have been estimated between 2% and 15% [1, 9, 16–19], with thse patiets facing a significantly increased risk of lung-related mortality [9–11, 20, 21]. Non-RA-ILD encompasses diverse pulmonary pathologies distinct from RA-ILD [14, 16], including obstructive lung diseases, with a reported prevalence ranging from 16 to 32% [22–26], organizing pneumonia (25 − 48%) [14, 16, 27], pleura involvment (up to 24%) [8, 12], drug-induced lug disese (1%) [28], and rheumatoid pulmonary nodules (< 1%) [28]. These complications add to the ovrall disease burden and contribute to increase mortality risks, in particular pneumonia and chronic obstructive diseases [28].

The occurrence of pulmonary extraarticular manifestations, while extensively studied in RA, is also a significant concern in patients with PsA. However, the reported prevalence of pulmonary involvement in PsA varies across studies from 0.5 to 9.4% [29–32]. Although less common than in RA, pulmonary complications such as pneumonia and chronic obstructive lung diseases are not only recognized as the second leading cause of death among PsA patients [4, 33], but also add to the substantial economic burden associated with PsA, further emphasizing the need for timely detection and management of such complications [34, 35]. 

Despite the impact of pulmonary manifestations in both RA and PsA, there remains a gap in understanding the various patterns of these manifestations, the factors contributing to their development, and their progression over time. However, early detection of lung involvement is crucial for enabling timely intervention, mitigating long-term complications, and improving the overall prognosis for patients with RA and PsA [36]. As pulmonary changes often precede clinical symptoms [8, 17], routine screening offers a unique opportunity to intervene at an earlier stage, potentially preventing irreversible damage [15]. However, there is a lack of consensus on the optimal methods and frequency of screening for pulmonary involvement in arthritis patients, highlighting the need for studies that evaluate the utility of various diagnostic tools both at baseline and in longitudinal follow-up.

In this context, a recent study by our group examined pulmonary involvement in newly diagnosed, untreated RA and PsA patients, revealing critical gaps in knowledge [13]. Thus, there was a notable discrepancy between respiratory symptoms and the findings from pulmonary function tests (PFTs) as well as imaging with chest radiography (CXR). Given the substantial number of patients without clinical symptoms, it is crucial to identify these asymptomatic high-risk individuals through suitable screening methods. Moreover, longitudinal changes in PFTs and the reversibility of pulmonary pathology following treatment initiation in newly diagnosed RA and PsA patients remain unexplored.

This prospective, longitudinal observational study builds upon the findings of our baseline research [13] by focusing on the progression and dynamics of pulmonary involvement in patients with newly diagnosed and untreated RA and PsA after treatment initiation. Utilizing a multimodal approach that combines clinical and laboratory assessments, chest radiography (CXR), and PFT, we aim to track changes in pulmonary involvement after treatment initiation. Additionally, this study seeks to identify biomarkers that may predict disease progression and evaluate the utility of various diagnostic tools in monitoring pulmonary health in RA and PsA patients. By addressing these objectives, we aim to contribute to the development of a comprehensive screening and monitoring algorithm for pulmonary complications in arthritis patients, enabling earlier intervention and improved patient outcomes.

## Methods

### Patient characteristics

Newly diagnosed and untreated patients with RA and PsA were prospectively enrolled in the Department of Rheumatology at the University Hospital Bonn, Germany, from August 1, 2018, to August 31, 2022. The longitudinal observational study design included an initial baseline assessment, with follow-up visits scheduled at three, six, nine and twelve months (t3, t6, t9, t12). Diagnoses were made by a board-certified rheumatologist. Classification criteria applied were ACR/European League Against Rheumatism criteria of 2010 for RA [37], and CASPAR criteria for PsA [38]. Inclusion criteria required participants to possess the necessary physical and mental capacity for participation as well as age over 18 years. Exclusion criteria were defined as the use of any rheumatological or immunosuppressive medications. Age-, gender-, and disease history-matched control patients without a history of rheumatological conditions were also recruited from the same department.

### Clinical and laboratory assessment

Demographic data such as age, gender, and body mass index, along with detailed disease history for each patient, were recorded. Additionally, patients’ smoking history, pre-existing pulmonary diseases, current medications, and present symptoms were thoroughly evaluated. Patients with RA and PsA exhibiting respiratory symptoms, such as cough and/or dyspnea, were categorized as symptomatic.

At the baseline assessment and each subsequent follow-up visit, patients underwent a comprehensive physical examination by a board-certified rheumatologist as well as a laboratory assessment. This examination included lung auscultation, assessment of chest excursion, and analysis of breathing width. Pathological thresholds were set at a breathing width of < 3.0 cm and a chest excursion of < 8.0 cm. The extent of arthritis activity was evaluated through the duration of arthritic symptoms and the Disease Activity Score in 28 joints using C-reactive protein (DAS28CRP). Routine laboratory measurements conducted included C-reactive protein (CRP) levels, hematological counts, rheumatoid factor (RF) titers, anti-citrullinated peptide antibodies (ACPA), and N-terminal pro-B-type natriuretic peptide levels. Furthermore, a blood gas analysis was performed.

### Functional assessment

All arthritis patients underwent CXR at the time of diagnosis, prior to the administration of any treatment. CXR were analyzed by a board-certified radiologist with a focus on interstitial lung diseases from the Department of Radiology at the University Hospital Bonn (DK). CXR findings were subsequently categorized into three categories: (1) Patients exhibiting no pathological findings. (2) Patients indicative of pulmonary involvement in association with their arthritis. (3) Patients presenting with other findings.

Comprehensive PFT, a 6-minute walking test, and transthoracic echocardiography were conducted at baseline and subsequently repeated during each follow-up visit (t3, t6, t9, t12). For PFT, spirometry and body plethysmography were utilized. Vital capacity (VC), forced vital capacity (FVC), total lung capacity (TLC), forced expiratory volume during the first second of FVC (FEV1), Tiffeneau-index (FEV1/ FVC), and residual volume (RV) were assessed. Additionally, we measured the diffusing capacity for carbon monoxide (DLCO) using the single-breath technique. PFT results were categorized into three distinct types: (1) Obstructive disorder, characterized by an FEV1/FVC ratio below the lower limit of normal (LLN) without restrictive patterns; (2) Restrictive disorder, indicated by a TLC below the LLN, with a normal Tiffeneau-index; (3) Diffusional capacity disorder, defined by a DLCO less than 60.0%. In the six-minute-walking test (6MWT), the total distance covered by the participants within six minutes was measured. Transthoracic echocardiography was performed using 2-dimensional, M-mode, and Doppler techniques, providing a comprehensive evaluation of cardiac dimensions, ejection fraction, heart valve functionality and morphology, including any valvular regurgitation or stenosis, blood flow patterns, and the presence of pericardial effusion.

### Statistical analysis

In the descriptive statistical analysis, qualitative variables were summarized as counts and percentages, while quantitative variables were expressed as mean ± standard deviation (SD) along with the 95% confidence interval or as median with range, based on their distribution’s adherence to normality as assessed by the Kolmogorov-Smirnov test. Linear mixed models were applied for the examination of longitudinal changes in arthritic disease activity. Mixed logistic regression models were utilized to explore the association between restrictive lung abnormalities, arthritic disease activity, and rheumatological medication use. The results are presented as odds ratios with 95% confidence intervals. Statistical significance was considered at *p* < .05. All analyses were conducted using IBM SPSS 29.0 for Windows and R Commander.

### Ethical approval

The study was conducted in accordance with the Declaration of Helsinki and has been reviewed and approved by the ethics committee of the University Hospital Bonn, Germany (reference number: 209/18). Written informed consent was obtained from each patient prior to inclusion in the study.

## Results

### Patient characteristics

The study prospectively enrolled 50 patients, including 26 RA and 24 PsA cases, with a gender distribution of 27 males and 23 females. The control group consisted of 26 patients, 12 males and 14 females. Detailed demographics and characteristics are shown in Table 1.

**Table 1:** Demographic and clinical data. Table 1 presents the epidemiological and disease-related baseline characteristics of the study population, according to their rheumatological diagnosis. Abbr.: NYHA: New York Heart Association, BMI: Body Mass Index

		Diagnosis		
		Rheumatoid arthritis (*n* = 26)	Psoriatic arthritis (*n* = 24)	Control patients (*n* = 26)
**Age** **[years]**	**Mean**	52.7	46.3	48.4
	**Standard deviation**	16.1	12.8	11.9
**Sex**	**Female**	9	14	14
		(34.6%)	(58.3%)	(53.8%)
	**Male**	17	10	12
		(65.4%)	(41.7%)	(46.2%)
**BMI** **[kg/m** ^**2**^ **]**	**Mean**	25.1	26.9	27.0
	**Standard deviation**	4.1	5.3	4.1
**Smoking**	**Neversmoker**	17	14	19
		(65.4%)	(58.3%)	(73.1%)
	**Eversmoker**	9	10	7
		(34.6%)	(41.7%)	(26.9%)
**Chronic cough**	**No**	16	19	24
		(61.5%)	(79.2%)	(92.3%)
	**Yes**	10	5	2
		(38.5%)	(20.8%)	(7.7%)
**Dyspnea**	**no (NYHA I)**	20	19	24
		(76.9%)	(79.2%)	(92.3%)
	**Yes (NYHA II+)**	6	5	2
		(23.1%)	(20.8%)	(7.7%)

### Clinical and laboratory assessment

At baseline, a significantly reduced pathological breathing width was observed in 64.0% of arthritis patients (RA 69.2%, PsA 58.3%), compared to 23.1% observed in the control group (*p* < .001). No differences were found in thoracic excursion (*p* < .642) or lung auscultation (*p* =.544) between the arthritis patients and control subjects. Respiratory symptoms were present in 36.0% of the arthritis patient group (RA 42.3%, PsA 29.2%) and in 11.5% of the control group (*p* =.031). CRP levels exceeding 3 mg/l were found in 66.0% of arthritis patients (RA 69.2%, PsA 62.5%), significantly higher than the 13.6% observed in the control subjects (*p* < .001). Elevated CRP did not correlate significantly with radiographic pulmonary involvement (*p* =.117). The mean DAS28CRP was 4.0 (± 1.2; 95% CI: 3.5–4.4) in RA patients and 3.3 (± 1.1; 95% CI: 2.9–3.8) in PsA patients. Arthritis patients with radiographic pulmonary involvement showed a higher DAS28CRP (median 3.9, range 2.8) than those without (median 3.1, range 4.9) (*p* =.114). Increased RF levels (> 14 IU/ml) were found in 33.3% of arthritis patients (*p* =.026) and more frequent in subjects presenting with pulmonary manifestation on CXR (*p* =.024). ACPA levels exceeding 8 U/ml were found in 17.8% of arthritis patients, but not in control subjects (*p* =.095), without significant correlation to pulmonary anomalies on CXR (*p* = 1.000). Detailed results are shown are shown in Table 1. For a comprehensive overview of the rheumatological treatments administered throughout the study, detailed information is provided in sTable 2.

**Fig. 1:**
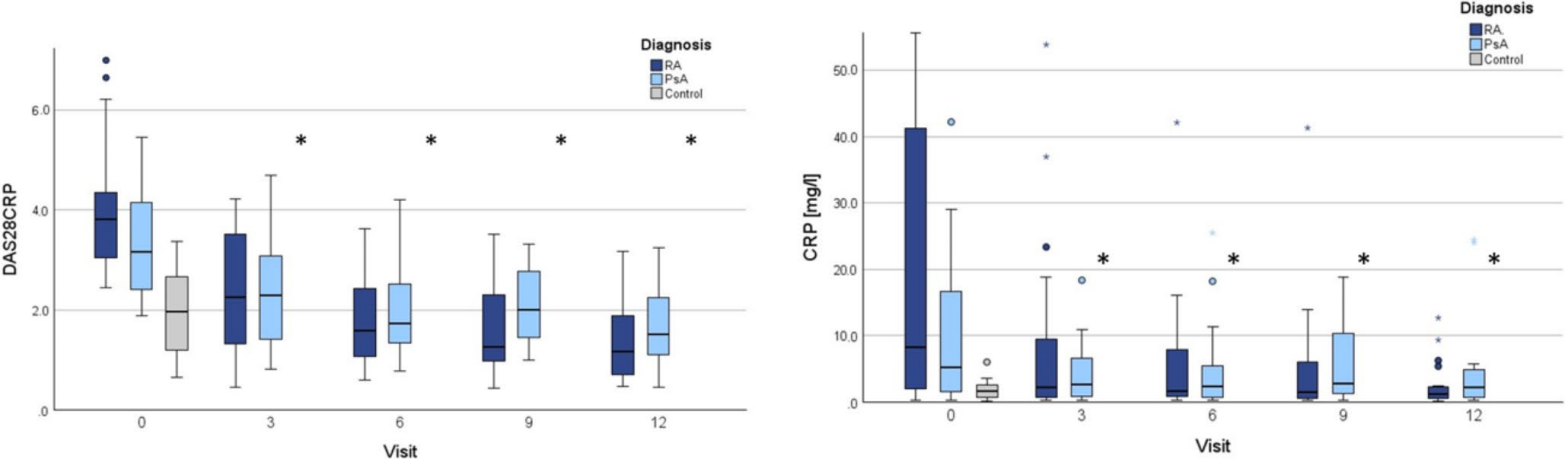
Longitudinal analysis of C-reactive protein levels and disease activity in rheumatoid arthritis and psoriatic arthritis patients. Figure 1 depicts the progression of C-reactive protein levels and Disease Activity Score in 28 joints using C-reactive protein in patients with rheumatoid arthritis and psoriatic arthritis. The data, captured at different time points (T0, T3, T6, T9, T12) following treatment initiation, demonstrates a significant reduction in both C-reactive protein levels and Disease Activity Score across both patient groups over the course of the study. Abbr.: SD: standard deviation, CI: confidence interval, RA: rheumatoid arthritis, PsA: psoriatic arthritis. *p < .05 was considered significant

During the follow-up period of our study, the results of the physical examinations, including auscultation, chest excursion, and breathing width, showed no significant changes over time in the arthritis patient group. However, there was a significant linear correlation between follow-up time and the occurrence of respiratory symptoms (*p* < .001). Moreover, a notable change was observed in the laboratory assessment. In particular, there was a significant reduction in CRP levels among arthritis patients, especially within the first three months following treatment initiation (*p* < .001). From visit T0 to visit T12, the mean CRP levels declined in RA patients from 23.5 mg/l (± 33.6; 95% CI: 9.9–37.1) to 2.7 mg/l (± 3.4; 95% CI: 1.0–4.3) and in PsA patients from 13.3 mg/l (± 18.0; 95% CI: 5.7–20.9) to 8.1 mg/l (± 16.2; 95% CI: 0.1–16.2). There was also a decrease in the disease course regarding arthritic disease activity, as measured by DAS28CRP (*p* < .001). The mean DAS28CRP declined in RA patients from 4.0 (± 1.2; 95% CI: 3.5–4.4) to 1.4 (± 0.8; 95% CI: 1.0–1.8) and in PsA patients from 3.3 (± 1.1; 95% CI: 2.9–3.8) to 1.6 (± 0.8; 95% CI: 1.25–2.0) (Table 2; Fig. 1).

**Table 2:** Analysis of C-reactive protein levels and disease activity in rheumatoid arthritis and psoriatic arthritis patients over time. Table 5 provides a detailed overview of the longitudinal changes in C-reactive protein levels and Disease Activity Score in 28 joints using C-reactive protein in patients with rheumatoid arthritis and psoriatic arthritis. The data is presented at various time points (T0, T3, T6, T9, T12) post-treatment initiation. A marked decrease in CRP levels and DAS28CRP scores is observed over time in both disease entities. Abbr.: SD: standard deviation, CI: confidence interval; *p < .05 was considered significant

C-reactive protein (mg/l)						
Visit	Mean (± SD)		Estimates		CI	*p*-value
	RA	PsA	RA	PsA		
**T0**	23.5 (± 33.6)	13.3 (± 18.0)	23.5	13.3	14.2–23.0	< 0.001*
**T3**	8.2 (± 13.3)	4.6 (± 4.8)	-15.3	-8.7	-18.4 – -6.0	< 0.001*
**T6**	5.5 (± 9.3)	4.7 (± 6.8)	-18.0	-8.6	-20.0 – -7.3	< 0.001*
**T9**	5.3 (± 9.0)	6.2 (± 7.0)	-18.2	-7.1	-19.4 – -6.6	< 0.001*
**T12**	2.7 (± 3.4)	8.1 (± 16.2)	-20.8	-5.2	-20.0 – -6.9	< 0.001*
**Disease Activity Score in 28 joints using C-reactive protein (DAS28CRP)**						
**Visit**	**Mean (± SD)**		**Estimates**		**CI**	**p-value**
	**RA**	**PsA**	**RA**	**PsA**		
**T0**	4.0 (± 1.2)	3.3 (± 1.1)	4.0	3.3	3.4–3.9	< 0.001*
**T3**	2.3 (± 1.2)	2.4 (± 1.1)	-1.7	-0.9	-1.7 – -1.0	< 0.001*
**T6**	1.8 (± 0.9)	2.0 (± 0.9)	-2.2	-1.3	-2.2 – -1.5	< 0.001*
**T9**	1.6 (± 0.9)	2.1 (± 0.8)	-2.4	-1.2	-2.2 – -1.5	< 0.001*
**T12**	1.4 (± 0.8)	1.6 (± 0.8)	-2.6	-1.7	-2.5 – -1.8	< 0.001*

### Functional assessment and chest radiography

**Fig. 2:**
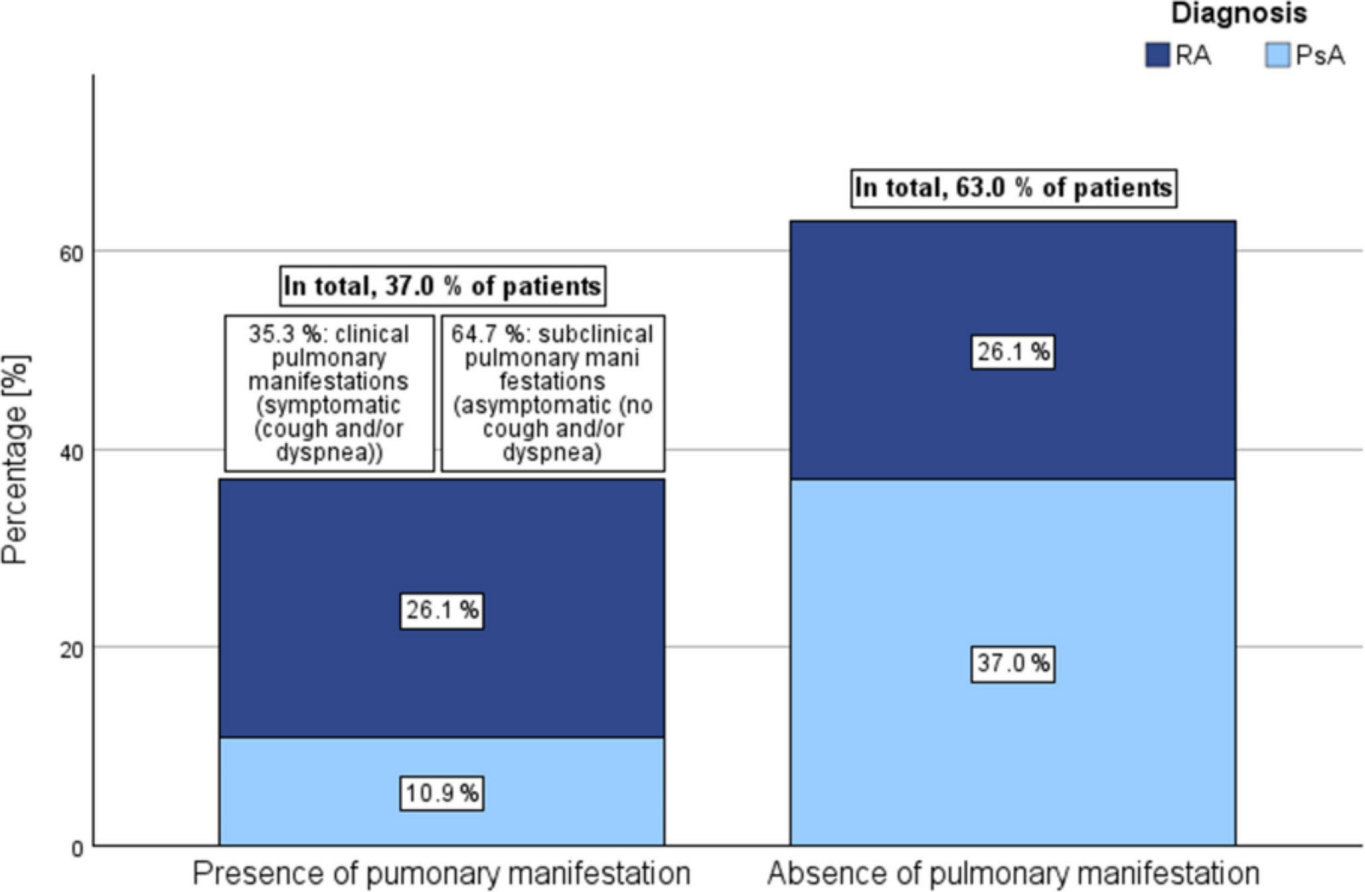
Correlation of radiographic pulmonary involvement with clinical symptoms in rheumatoid arthritis and psoriatic arthritis patients. Figure 2 displays the presence of radiographic pulmonary involvement and its association with clinical symptoms in patients with rheumatoid arthritis and psoriatic arthritis. Abbr. RA: rheumatoid arthritis, PsA: psoriatic arthritis

At baseline, radiographic evidence of pulmonary involvement, as detected through CXR, was observed in 37.0% of arthritis patients, specifically 50.0% of RA cases and 22.7% of PsA cases (*p* =.072). Within the cohort of arthritis patients demonstrating radiographic pulmonary involvement on CXR, 35.3% were symptomatic, whereas 64.7% remained asymptomatic (Fig. 2). Baseline PFT identified restrictive ventilatory disorders in 19.0% of arthritis patients (RA 27.3%, PsA 10.0%) and 24.0% of control subjects (*p* =.758), with no obstructive patterns observed. No significant association was found between radiographic pulmonary involvement and the following pulmonary function parameters: FVC (*p* =.344), TLC (*p* =.245), FEV1 (*p* =.740), Tiffeneau-index (*p* =.976), and DLCO (*p* =.654). Detailed data for PFT over time are presented in sTable 1. Transthoracic echocardiographic evaluations revealed no considerable cardiac abnormalities or association with pulmonary involvement (data not shown). The average 6MWT distance did not demonstrate significant differences concerning arthritis diagnosis or radiographic pulmonary involvement (data not shown).

**Fig. 3:**
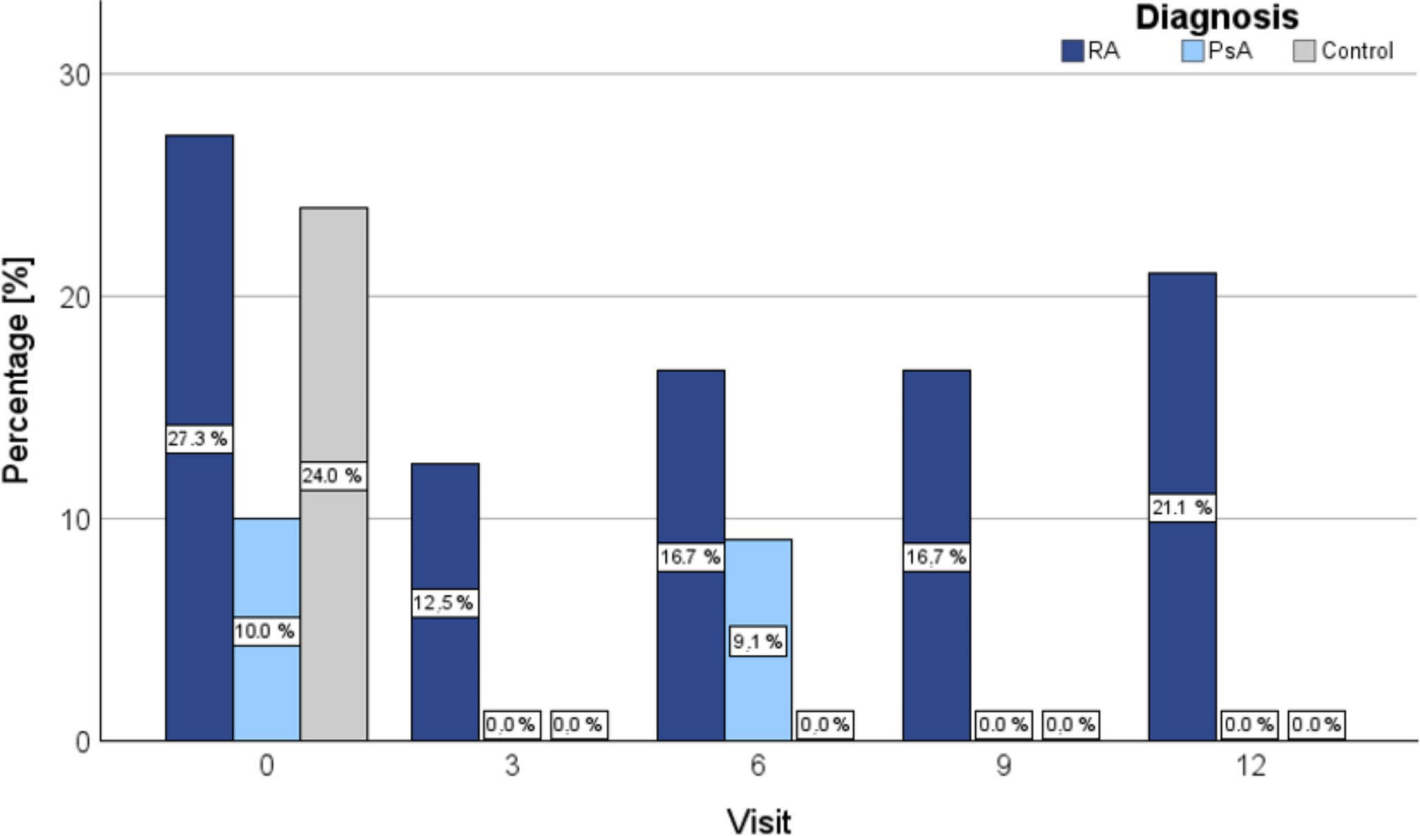
Dynamic trends in restrictive ventilatory disorders in rheumatoid arthritis and psoriatic arthritis patients over time. Figure 3 illustrates the progression of restrictive ventilatory disorders in patients diagnosed with rheumatoid arthritis and psoriatic arthritis over a series of time points (T0, T3, T6, T9, T12) following the initiation of treatment Notably, rheumatoid arthritis patients show a marked initial decline in the prevalence of restrictive ventilatory disorders, followed by a gradual increase. Conversely, psoriatic arthritis patients exhibit a trend towards the resolution of these disorders over time. Abbr.: SD: standard deviation, CI: confidence interval, RA: rheumatoid arthritis, PsA: psoriatic arthritis; *p < .05 was considered significant

**Fig. 4:**
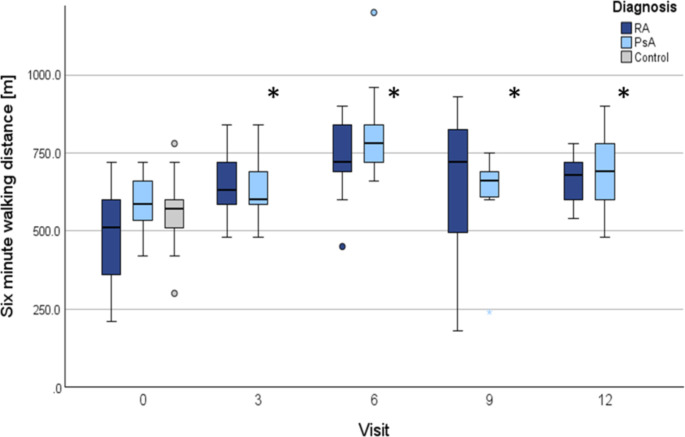
**Six-minute-walking distance in rheumatoid arthritis and psoriatic arthritis patients over time.** Figure 4 depicts the progression of six-minute-walking distance in patients with rheumatoid arthritis and psoriatic arthritis. The data, captured at different time points (T0, T3, T6, T9, T12) following treatment initiation, demonstrates a significant increase in the six-minute walking distance across both patient groups over the course of the study. Abbr.: SD: standard deviation, CI: confidence interval, RA: rheumatoid arthritis, PsA: psoriatic arthritis; *p < .05 was considered significant

During the follow-up period, the mean values of FVC, TLC, Tiffeneau index, RV, and DLCO remained stable with no significant changes over time, and no significant change was observed in obstructive ventilatory disorders, nor prevalence of emphysema or diffusion disorders. However, the incidence of restrictive lung disease decreased over the follow-up period: from 19.0% (RA 27.3%, PsA 10.0%) at baseline (t0) to 12.5% (RA 21.1%, PsA 0.0%) at twelve months (*p* =.373) (Fig. 3). No significant associations were identified between the DAS28-CRP score and restrictive lung disease (*p* =.700) or between CRP levels and restriction (*p* =.301) throughout the disease course. Similarly, the analysis of smoking, BMI, and symptom duration revealed no significant impact on restrictive lung disease across visits. BMI (*p* =.256), symptom duration (*p* =.437), and pack-years (*p* =.804) did not significantly affect the occurrence of pulmonary restriction over time. No significant association was found regarding the switch to bDMARDs and tsDMARDs and the presence of restrictive lung disease (*p* =.129). Total walking distance in the 6MWT showed overall improvement compared to baseline at all follow-up visits (*p* < .001). From visit T0 to visit T12, the mean walking distance improved in RA patients from 491.4 m (± 155.0; 95% CI: 401.9–580.9) to 675.6 m (± 83.5; 95% CI: 611.3 − 739.8) and in PsA patients from 587.1 m (± 92.2; 95% CI: 528.5 − 645.6) to 687.0 m (± 130.0; 95% CI: 594.0 − 780.0). Detailed results are shown in Table 3; Fig. 4.

**Table 3:** Progression of walking distance in rheumatoid arthritis and psoriatic arthritis patients over time. Table 6 illustrates the longitudinal changes in walking distance (measured in meters) at different time points (T0, T3, T6, T9, T12) in patients with rheumatoid arthritis and psoriatic arthritis. Notable improvements in walking distance can be observed, with significant increases at T3, T6, T9, and T12. Abbr.: SD: standard deviation, CI: confidence interval; *p < .05 was considered significant

Walking distance [m]						
Visit	Mean (± SD)		Estimates		CI	*p*-value
	RA	PsA	RA	PsA		
**T0**	491.4 (± 155.0)	587.1 (± 92.2)	491.4	587.1	477.9–588.5	< 0.001*
**T3**	645.0 (± 102.8)	632.7 (± 102.6)	+ 153.9	+ 45.6	17.9–164.0	0.015*
**T6**	741.8 (± 136.8)	820.0 (± 172.3)	+ 250.7	+ 232.9	147.9–296.8	< 0.001*
**T9**	624.5 (± 249.5)	613.8 (± 158.6)	+ 133.4	+ 26.7	5.3–156.7	0.036*
**T12**	675.6 (± 83.5)	687.0 (± 130.0)	+ 184.5	+ 99.9	81.1–229.0	< 0.001*

## Discussion

This study represents a pioneering investigation into pulmonary involvement in newly diagnosed RA and PsA patients, with a one-year follow-up after treatment initiation. Adopting a comprehensive longitudinal approach, it aimed to assess the utility of various diagnostic modalities in monitoring pulmonary changes and to identify potential biomarkers indicative of pulmonary disease progression.

At baseline, our findings indicated a 37.0% incidence of radiographic pulmonary involvement in patients newly diagnosed with RA and PSA, as previously reported [13]. Specifically, 50.0% o RA patients and 22.7% o PsA patients demonstrated radiographic evidence of pulmonary involvement. Prior prospective studies on pulmonary complications in arthritis predominantly focused on RA, showing a wide range of prevalence rates [6, 20, 39, 40]. For instance, while some studies reported as low as a 10.0% prevalence of pumonary involvement in RA [40], others, like the study by Bilgici et al. employing high-resolution computed tomography and pulmonary function tests, noted abnormalities in up to 67.3% of RA cases [6]. While te baseline findings of our study on RA align with prior research, we observed a significantly higher prevalence of pulmonary involvement in PsA patients than the previously reported 0.5 to 9.4% [29–32]. The detected revalenc rates at diagnosis for RA and PsA highlight the urgent need for increased awareness, proactive screening, and regular follow-up even in early-stage patients. Moreover, the fact that most patients with pulmonary involvement were asymptomatic necessitates a reevaluation of existing screening protocols to prevent delayed diagnosis and treatment, which could negatively impact patient outcomes. Our study indicates that clinicians should be alert to indicators like high disease activity as measured by DAS28CRP, increased age, and elevated rheumatoid factor levels, even in the absence of symptoms like dyspnea and cough [13].

However, further research is needed to understand not only the initial pulmonary involvement but also its long-term progression and the impact of treatment initiation. Previous studies have mainly utilized imaging techniques to monitor pulmonary progression in RA and PsA, but these methods are limited due to radiation exposure concerns. Our research expands on this by incorporating PFT as an alternative, radiation-free diagnostic method to track longitudinal changes in pulmonary disease progression and to assess the potential reversibility of pulmonary involvement with therapy initiation. Few studies have used PFT in RA [6, 21, 39, 41–44], and these have highlighted a significant risk for RA patients, especially those with newly diagnosed disease (≤ 2 years), who face a tenfold increased risk of rapid lung function decline, as indicated by a decrease in FVC [39]. Both reduced FVC and high disease activity are independently associated with increased mortality [45]. The utility of PFT in assessing pulmonary involvement in PsA, however, has not yet been explored, rendering our research a pioneering effort.

The absence of a significant association between PFT results, including the 6MWT, and CXR findings at baseline underscores the complexity of pulmonary involvement in RA and PsA. It suggests that early radiographic and functional changes in the lungs may not immediately translate into measurable changes in PFT outcomes. This observation is consistent with the fact that while bodyplethysmography is a suitable and widely available tool for assessing the clinical relevance of lung involvement, it lacks sensitivity, particularly for early and subclinical lesions [41]. Prior studies have not found a significant link between various PFT parameters and lung involvement as detected by high-resolution computed tomography in RA patients [6, 43, 44]. Our findings align with these studies, indicating that while PFT is valuable for evaluating overall lung function and identifying more advanced lung disease, it may not be as effective in detecting early-stage or mild pulmonary involvement. Therefore, it is plausible that some cases of lung involvement may go undetected when relying solely on PFT results, potentially leading to an underestimation of prevalence rates.

During the follow-up period of our study, most functional assessment outcomes, including clinical and laboratory assessments, remained stable. There were no significant changes over time in mean values for FVC, TLC, Tiffeneau index, RV, and DLCO. Additionally, no notable changes were observed in obstructive ventilation disorders. However, the decline in the incidence of restrictive lung disease during the follow-up is noteworthy, despite the lack of an association between arthritis disease activity and the risk of developing restrictive lung disorders. This observation, as well as our baseline results, shows no association between the level of disease activity (as indicated by CRP levels and DAS28CRP score) and the outcomes of PFT, DLCO, or 6MWT, questions an association between disease activity and pulmonary involvement. Our findings suggest that reducing arthritic disease activity does not necessarily reduce the risk or severity of pulmonary involvement.

The existing literature on the relationship between disease activity and pulmonary involvement presents mixed findings. Some studies have found a significant connection between radiographic lung involvement (diagnosed by high-resolution computed tomography) and disease activity (measured by DAS28CRP) [6, 43, 46], and have also linked PFT results to disease activity [42]. In contrast, other research, including a large prospective cohort study, did not find a correlation between DAS28CRP and the risk of pulmonary involvement diagnosed by high-resolution computed tomography, nor between PFT results and DAS28CRP [20, 39]. In our study, smoking, BMI, and symptom duration showed no significant associations with pulmonary function across study visits. These findings partially contradict existing literature, which identifies smoking history and arthritis symptom duration exceeding five months as risk factors for interstitial lung disease in rheumatoid arthritis [47]. The lack of significant associations in our study may be attributable to the relatively small sample size. Moreover, pulmonary changes in arthritis patients may develop over extended periods, highlighting the need for longer follow-up or more sensitive pulmonary assessments to capture these effects. This underscores the complexity of pulmonary involvement in arthritis and the necessity for further research to better understand its multifactorial etiology.

The improvement in walking distance, as measured by the 6MWT, independent of disease activity, further suggests multifactorial influences on functional capacity. The outcomes of the 6MWT might be affected by a combination of factors beyond disease activity. These factors could include a reduction in arthritis-associated pain, leading to increased daily activity and mobility, which in turn might result in training effects improving overall functional capacity. Additionally, improvements in pulmonary capacities behavioral adaptations may contribute to the enhanced walking distances observed. Conversely, arthritis-associated pain, particularly when involving weight-bearing joints such as the knees, and limited mobility may adversely impact 6MWT performance. Our findings highlight that, while the 6MWT is a valuable tool for assessing functional capacity, its results in arthritis patients should be interpreted within the context of diverse influencing factors. These factors extend beyond disease activity and pulmonary involvement, emphasizing the complexity of functional assessments in this patient population.

Furthermore, our study found no significant association between rheumatological medications and PFT or 6MWT results, suggesting that early-stage medication choices may not have a substantial impact on lung function or capacity. Notably, our study did not detect evidence of potential drug-induced lung diseases within the observed timeframe. This absence of evidence offers some reassurance about the short-term pulmonary safety of these medications, but it is crucial to maintain vigilance for possible long-term pulmonary effects [48].

In summary, our study underscores the frequent occurrence of pulmonary involvement in newly diagnosed, untreated RA and PsA patients, many of whom display no symptoms. The considerable impact of pulmonary involvement on the quality of life, morbidity, and mortality in these patient groups emphasizes the need for thorough monitoring, particularly during the early years of disease activity and upon therapy modification. Although CXR, PFT with DLCO, and 6MWT emerge as potential diagnostic tools for initial assessment and tracking, our study revealed no significant changes over time, indicating the need for more sensitive and specific diagnostic tools.

While our research provides important insights into the pulmonary involvement of RA and PsA, it also highlights the need for further research with several limitations warranting consideration. Larger-scale studies with extended follow-up periods are essential to validate our findings and better understand the long-term evolution of pulmonary involvement and its responsiveness to therapy. Moreover, the relatively small sample size reduces the statistical power to detect subtle subgroup differences and limits the generalizability of our findings. Additionally, the diagnostic methods used also have inherent limitations. While chest radiography and pulmonary function tests are practical and widely accessible, they may lack the sensitivity needed to detect early or subtle pulmonary abnormalities. Advanced imaging techniques, such as HRCT, could offer more precise insights into the extent and nature of pulmonary changes.

Future studies could benefit from a more comprehensive assessment of disease manifestations, particularly when evaluating axial and peripheral disease involvement. The DAS28-CRP, while a valuable tool for assessing peripheral joint disease activity, may not adequately capture axial PsA manifestations, thereby limiting the understanding of their relationship with pulmonary involvement. Additionally, this study did not separately evaluate arthritis localization or the number of affected joints, both of which could provide critical insights into their potential impact on pulmonary function. Finally, the comparative approach in this study, analyzing RA and PsA side by side, offers important insights but also presents challenges. These diseases have distinct pathophysiological mechanisms, which may reduce the applicability of unified conclusions. Addressing these differences in future studies by tailoring methodologies to the unique features of each condition will improve the understanding of their respective pulmonary complications.

## Electronic Supplementary Material

Below is the link to the electronic supplementary material.

Supplementary Material 1

## Data Availability

The datasets collected for this manuscript are available from the corresponding author upon reasonable request.

## Declarations

### Patient and public involvement

Patients and/or the public were not involved in the design, or conduct, or reporting, or dissemination plans of this research.

### Note on related congress abstracts and publication

This manuscript is an original work and has not been published or submitted elsewhere, in part or in whole. A related publication addressed the baseline findings of this cohort: Schäfer VS, Winter L, Skowasch D, et al. *Exploring pulmonary involvement in newly diagnosed rheumatoid arthritis and psoriatic arthritis: A single-center stud*y. *Rheumatol Int.* 2024 Oct;44(10):1975–1986. doi: 10.1007/s00296-024-05685-3. Epub 2024 Aug 21. PMID: 39167172; PMCID: PMC11392970.

While the prior study focused on baseline examination results, the current manuscript presents a longitudinal analysis of follow-up data from the same cohort, specifically examining the progression of pulmonary involvement in patients with rheumatoid arthritis and psoriatic arthritis. A summary of baseline findings is included for contextual clarity, resulting in minor overlap with the previous publication. In cases of overlap, the prior study is appropriately cited to acknowledge its contribution.
