# Supplementary material for: Pulmonary involvement in newly diagnosed and untreated rheumatoid arthritis and psoriatic arthritis: a prospective longitudinal study
Source: Rheumatol Int. 2024 Dec 18;45(1):3. doi: 10.1007/s00296-024-05751-w (PMC11655587; doi:10.1007/s00296-024-05751-w)
Supplement: Supplementary file 1 — Supplementary Material 1 [file 296_2024_5751_MOESM1_ESM.docx]

**SUPPLEMENTARY MATERIAL:**

**sTables**

|  | | **Visit** | | | | | | | | | | |
| --- | --- | --- | --- | --- | --- | --- | --- | --- | --- | --- | --- | --- |
|  |  | **T0** | | | **T3** | | **T6** | | **T9** | | **T12** | |
|  |  | **RA**  **(n = 26)** | **PsA**  **(n = 24)** | **Control**  **(n = 26)** | **RA**  **(n = 25)** | **PsA**  **(n = 25)** | **RA**  **(n = 25)** | **PsA**  **(n = 25)** | **RA**  **(n = 25)** | **PsA**  **(n = 25)** | **RA**  **(n = 25)** | **PsA**  **(n = 25)** |
| **Respiratory symptomatic**  **(cough and/or dyspnea)** | Not present | 15  (57.7 %) | 17  (70.8 %) | 23  (88.5 %) | 20  (87.0 %) | 17  (85.0 %) | 19  (90.5 %) | 16  (88.9 %) | 20  (100.0 %) | 16  (100.0 %) | 18  (100.0 %) | 14  (93.3 %) |
|  | Present | 11  (42.3 %) | 7  (29.2 %) | 3  (11.5 %) | 3  (13.0 %) | 3  (15.0 %) | 2  (9.5 %) | 2  (11.1 %) | 0  (0.0 %) | 0  (0.0 %) | 0  (0.0 %) | 1  (6.7 %) |
| **Auscultation** | Vesicular breath sound | 26  (100.0 %) | 22  (91.7 %) | 26  (100.0%) | 24  (100.0 %) | 20  (100.0 %) | 19  (90.5 %) | 18  (100.0 %) | 20  (100.0 %) | 15  (93.8 %) | 18  (100.0 %) | 15  (100.0 %) |
|  | Wheezing/ Obstruction | 0  (0.0 %) | 2  (8.3 %) | 0  (0.0 %) | 0  (0.0 %) | 0  (0.0 %) | 2  (9.5 %) | 0  (0.0 %) | 0  (0.0 %) | 1  (6.3 %) | 0  (0.0 %) | 0  (0.0 %) |
| **Breathing width** | Non-pathological  (≥ 3 cm) | 8  (30.8 %) | 10  (41.7 %) | 20  (76.9 %) | 6  (26.1 %) | 7  (35.0 %) | 6  (28.6 %) | 8  (47.1 %) | 6  (30.0 %) | 6  (40.0 %) | 6  (33.3 %) | 6  (42.9 %) |
|  | Pathological  (< 3 cm) | 18  (69.2 %) | 14 (58.3 %) | 6  (23.1 %) | 17  (73.9 %) | 13  (65.0 %) | 15  (71.4 %) | 9  (52.9 %) | 14  (70.0 %) | 9  (60.0 %) | 12  (66.7 %) | 8  (57.1 %) |
| **Chest excursion** | Non-pathological  (≥ 8 cm) | 15  (57.7 %) | 14  (58.3 %) | 17  (65.4 %) | 16  (69.6 %) | 14  (70.0 %) | 17  (81.0 %) | 13  (76.5 %) | 20  (100.0 %) | 12  (80.0 %) | 18  (100.0 %) | 12  (85.7 %) |
|  | Pathological  (< 8 cm) | 11  (42.3 %) | 10  (41.7 %) | 9  (34.6 %) | 7  (30.4 %) | 6  /30.0 %) | 4  (19.0 %) | 4  (23.5 %) | 0  (0.0 %) | 3  (20.0 %) | 0  (0.0 %) | 2  (14.3 %) |
| **TLC [%]** | Mean | 94.8 | 101.3 | 95.3 | 97.9 | 105.5 | 99.7 | 106.7 | 97.3 | 105.6 | 94.3 | 106.0 |
|  | SD | 15.3 | 11.9 | 18.0 | 14.8 | 12.5 | 14.4 | 15.0 | 13.7 | 14.9 | 19.1 | 16.2 |
| **FVC [%]** | Mean | 90.0 | 95.3 | 90.8 | 91.8 | 95.0 | 89.9 | 94.3 | 86.4 | 99.8 | 90.9 | 98.8 |
|  | SD | 15.5 | 11.5 | 13.8 | 11.0 | 12.8 | 15.0 | 10.0 | 13.1 | 12.2 | 12.5 | 11.4 |
| **FEV1/FVC [%]** | Mean | 110.1 | 105.1 | 101.4 | 102.1 | 101.0 | 100.2 | 98.7 | 102.1 | 101.2 | 100.2 | 100.1 |
|  | SD | 13.9 | 13.5 | 10.0 | 5.0 | 7.8 | 6.2 | 8.5 | 5.7 | 7.3 | 7.1 | 7.7 |
| **RV [%]** | Mean | 104.6 | 98.3 | 99.2 | 117.9 | 118.1 | 111.7 | 126.7 | 116.6 | 111.0 | 111.8 | 108.8 |
|  | SD | 42.7 | 31.1 | 45.3 | 28.9 | 34.8 | 31.7 | 34.7 | 43.3 | 31.2 | 41.4 | 42.7 |
| **DLCO [%]** | Mean | 74.0 | 75.8 | 80.6 | 75.2 | 72.2 | 72.7 | 73.7 | 75.5 | 71.8 | 71.4 | 74.8 |
|  | SD | 13.4 | 13.6 | 17.0 | 13.3 | 8.6 | 13.9 | 5.5 | 11.4 | 9.9 | 12.1 | 10.2 |
| **pCO_2_ [mmHg]** | Mean | 36.4 | 35.6 | 35.7 | 38.1 | 35.8 | 37.7 | 37.5 | 36.3 | 35.9 | 36.3 | 33.5 |
|  | SD | 5.6 | 4.6 | 4.9 | 5.0 | 4.8 | 5.2 | 3.2 | 4.5 | 2.9 | 4.4 | 2.5 |
| **pO_2_ [mmHg]** | Mean | 82.9 | 79.5 | 78.6 | 72.6 | 73.3 | 76.9 | 75.8 | 79.9 | 79.6 | 76.1 | 78.2 |
|  | SD | 9.6 | 9.6 | 7.8 | 20.0 | 18.8 | 18.6 | 6.4 | 9.5 | 7.1 | 11.8 | 11.4 |
| **Hb [g/dl]** | Mean | 13.9 | 14.0 | 14.6 | 13.6 | 14.2 | 13.5 | 13.8 | 13.6 | 14.0 | 13.8 | 14.0 |
|  | SD | 1.2 | 1.4 | 1.4 | 1.3 | 1.3 | 1.3 | 1.3 | 1.2 | 1.3 | 1.3 | 1.3 |
| **CRP [mg/l]** | Mean | 23.5 | 13.3 | 6.6 | 8.2 | 4.6 | 5.5 | 4.7 | 5.3 | 6.2 | 2.7 | 8.1 |
|  | SD | 33.6 | 18.0 | 22.7 | 13.3 | 4.8 | 9.3 | 6.7 | 9.0 | 7.0 | 3.4 | 16.2 |
| **DAS28CRP** | Median | 3.8 | 3.2 | 2.0 | 2.3 | 2.3 | 1.6 | 1.7 | 1.3 | 2.0 | 1.2 | 1.5 |
|  | Range | 4.6 | 3.6 | 2.7 | 3.8 | 3.9 | 3.0 | 3.4 | 3.1 | 2.3 | 2.7 | 2.8 |
| **Six-minute walking distance [m]** | Mean | 491.4 | 587.1 | 560.3 | 645.0 | 632.7 | 741.8 | 820.0 | 624.5 | 613.8 | 675.6 | 687.0 |
|  | SD | 155.0 | 92.2 | 106.3 | 102.8 | 102.6 | 136.9 | 172.3 | 249.5 | 158.6 | 83.5 | 130.0 |

**sTable 1: Functional assessment in rheumatoid arthritis, psoriatic arthritis, and control group patients over time**

sTable 1 presents an extensive overview of respiratory symptoms, lung function tests, and functional assessments across patients with rheumatoid arthritis, psoriatic arthritis, and control patients at multiple time points (T0, T3, T6, T9, T12). Abbr.: T0-12: time of visit, RA: rheumatoid arthritis, PsA: psoriatic arthritis, SD: standard deviation, FVC: forced vital capacity, TLC: total lung capacity, FEV1: forced expiratory volume during the first second of FVC, RV: residual volume, DLCO: Diffusing capacity for carbon monoxide, pO_2_: partial pressure of oxygen, pCO_2_: partial pressure of carbon dioxide, Hb: hemoglobin, CRP: C-reactive protein, DAS28CRP: Disease Activity Score in 28 joints using CR

|  | | **Visit** | | | | | | | | | |
| --- | --- | --- | --- | --- | --- | --- | --- | --- | --- | --- | --- |
| **Rheumatological medication** | | **T0** | | **T3** | | **T6** | | **T9** | | **T12** | |
|  |  | **RA**  **(n = 26)** | **PsA**  **(n = 24)** | **RA**  **(n = 25)** | **PsA**  **(n = 25)** | **RA**  **(n = 25)** | **PsA**  **(n = 25)** | **RA**  **(n = 25)** | **PsA**  **(n = 25)** | **RA**  **(n = 25)** | **PsA**  **(n = 25)** |
| **Glucocorticoid** | | 22  (44.0 %) | 18  (36.0 %) | 10  (20.0 %) | 9  (18.0 %) | 10  (20.0 %) | 8  (16.0 %) | 6  (12.0 %) | 3  (6.0 %) | 5  (10.0 %) | 3  (6.0 %) |
|  | Mean dosage [mg/day] | 21.8 | 22.8 | 9.8 | 17.5 | 10.1 | 15.3 | 10.4 | 8.7 | 9.1 | 10.7 |
| **cs DMARD** | Methotrexate | 23 | 21 | 22 | 16 | 15 | 14 | 17 | 12 | 16 | 12 |
|  | Leflunomide | 0 | 0 | 2 | 2 | 2 | 0 | 2 | 0 | 0 | 0 |
|  | Hydroxy-chloroquine | 0 | 0 | 0 | 0 | 1 | 0 | 1 | 0 | 1 | 0 |
|  | Sulfasalazine | 1 | 0 | 1 | 0 | 3 | 0 | 3 | 0 | 3 | 0 |
|  |  | 45  (90.0 %) | | 40  (80.0 %) | | 35  (70.0 %) | | 35  (70.0 %) | | 31  (62.0 %) | |
| **ts**  **DMARD** | Apremilast | 0 | 1 | 0 | 2 | 0 | 3 | 0 | 1 | 0 | 1 |
|  | Baricitinib | 0 | 1 | 1 | 0 | 3 | 0 | 2 | 0 | 2 | 0 |
|  | Tofacitinib | 0 | 0 | 0 | 0 | 0 | 2 | 0 | 2 | 0 | 1 |
|  | Upadacitinib | 0 | 0 | 0 | 0 | 0 | 0 | 0 | 0 | 1 | 1 |
|  |  | 2  (4.0 %) | | 3  (6.0 %) | | 8  (16.0 %) | | 5  (10.0 %) | | 6  (12.0 %) | |
| **b**  **DMARD** | TNF alpha inhibitor | 0 | 2 | 0 | 5 | 0 | 4 | 2 | 5 | 3 | 5 |
|  | Abatacept | 0 | 0 | 0 | 0 | 0 | 0 | 1 | 0 | 1 | 0 |
|  | Secukinumab | 0 | 0 | 0 | 0 | 0 | 1 | 0 | 1 | 0 | 2 |
|  |  | 2  (4.0 %) | | 5  (10.0 %) | | 5  (10.0 %) | | 8  (16.0 %) | | 11  (22.0 %) | |

**sTable 2: Rheumatological medicaiton usage in rheumatoid arthritis and psoriatic arthritis patients over time**

sTable 2 displays rheumatological medications among patients with rheumatoid arthritis and psoriatic arthritis over five time points (T0, T3, T6, T9, T12). Abbr.: csDMARD: conventional synthetic disease-modifying anti-rheumatic drug, tsDMARD: targeted synthetic disease-modifying anti-rheumatic drug, bDMARD: biological disease-modifying anti-rheumatic drug, TNF: tumor necrosis factor
